# Highly Repetitive Genome of *Coniella granati* (syn. *Pilidiella granati*), the Causal Agent of Pomegranate Fruit Rot, Encodes a Minimalistic Proteome with a Streamlined Arsenal of Effector Proteins

**DOI:** 10.3390/ijms25189997

**Published:** 2024-09-17

**Authors:** Antonios Zambounis, Elisseos I. Maniatis, Annamaria Mincuzzi, Naomi Gray, Mohitul Hossain, Dimitrios I. Tsitsigiannis, Epaminondas Paplomatas, Antonio Ippolito, Leonardo Schena, James K. Hane

**Affiliations:** 1 Hellenic Agricultural Organization—DIMITRA (ELGO—DIMITRA), Institute of Plant Breeding and Genetic Resources, 57001 Thessaloniki, Greece; 2 Laboratory of Plant Pathology, Department of Crop Science, Agricultural University of Athens, 11855 Athens, Greece; maniatis.elisseos@gmail.com (E.I.M.); dimtsi@aua.gr (D.I.T.); epaplom@aua.gr (E.P.); 3 Department of Soil, Plant and Food Sciences, University of Bari Aldo Moro, 70126 Bari, Italy; annamaria.mincuzzi@uniba.it (A.M.); antonio.ippolito@uniba.it (A.I.); 4 Centre for Crop and Disease Management, Department of Molecular and Life Sciences, Curtin University, Bentley, Perth 6102, Australia; naomi.gray@postgrad.curtin.edu.au (N.G.); mdmohitul.hossain@postgrad.curtin.edu.au (M.H.); 5 Department of Agriculture, Mediterranea University of Reggio Calabria, 89124 Reggio Calabria, Italy; lschena@unirc.it

**Keywords:** *Coniella granati*, pomegranate, Sordariomycetes, plant–pathogen interactions, pathogenicity effectors

## Abstract

This study describes the first genome sequence and analysis of *Coniella granati*, a fungal pathogen with a broad host range, which is responsible for postharvest crown rot, shoot blight, and canker diseases in pomegranates. *C. granati* is a geographically widespread pathogen which has been reported across Europe, Asia, the Americas, and Africa. Our analysis revealed a 46.8 Mb genome with features characteristic of hemibiotrophic fungi. Approximately one third of its genome was compartmentalised within ‘AT-rich’ regions exhibiting a low GC content (30 to 45%). These regions primarily comprised transposable elements that are repeated at a high frequency and interspersed throughout the genome. Transcriptome-supported gene annotation of the *C. granati* genome revealed a streamlined proteome, mirroring similar observations in other pathogens with a latent phase. The genome encoded a relatively compact set of 9568 protein-coding genes with a remarkable 95% having assigned functional annotations. Despite this streamlined nature, a set of 40 cysteine-rich candidate secreted effector-like proteins (CSEPs) was predicted as well as a gene cluster involved in the synthesis of a pomegranate-associated toxin. These potential virulence factors were predominantly located near repeat-rich and AT-rich regions, suggesting that the pathogen evades host defences through Repeat-Induced Point mutation (RIP)-mediated pseudogenisation. Furthermore, 23 of these CSEPs exhibited homology to known effector and pathogenicity genes found in other hemibiotrophic pathogens. The study establishes a foundational resource for the study of the genetic makeup of *C. granati*, paving the way for future research on its pathogenicity mechanisms and the development of targeted control strategies to safeguard pomegranate production.

## 1. Introduction

*Coniella granati* (Sacc.) Petr. & Syd [syn. *Pilidiella granati* (Sacc.)] [1] is a ubiquitous fungal pathogen with a global distribution, which poses a significant economic threat to pomegranate (*Punica granatum* L.) production. It causes various symptoms in different host tissues, including crown rot (also known as ‘pomegranate fruit rot’ or Coniella rot) [2,3], shoot blight (‘leaf blotch of pomegranate’), dieback, and canker diseases. Reports of its impact span continents [4], including from Europe [1,5,6,7,8,9,10,11], the Middle East [12,13], Asia [14,15,16], the Americas [17,18], and Africa [19,20]. Despite its widespread impact, a comprehensive understanding of the precise epidemiology of this thermophilic pathogen remains elusive [21].

*C. granati* attacks the fruit of pomegranate trees, causing fruit softening. Following its initial infection, *C. granati* can remain latent for several months until the fruit ripens or even later [2,22,23]. This cryptic presence delays detection until the postharvest stage, often resulting in significant losses due to rapid fruit deterioration. Artificially infected fruits rot within 11 to 15 days after pathogen inoculation, resulting in substantial losses [24]. Recently, the transcriptional reprogramming of pomegranate fruit upon pathogen inoculation was deciphered using a time series at three different periods after inoculation [25].

While *C. granati* is recognised as a major pathogen of pomegranate [17], it also demonstrates a broader host range. Reports indicate that it is able to infect a diverse range of plant species, including ornamental roses (*Rosa* spp.) [26], citrus trees (*Citrus* spp.) [27], grapevines (*Vitis vinifera*) [28], rubber plants (*Hevea* spp.) [1], Indian beech (*Anogeissus acuminata*) [29], and the red bird of paradise (*Caesalpinia pulcherrima*) [30].

Despite the threat posed by *C. granati*, fungicide efficacy testing has so far been limited to in vitro studies. These investigations have explored a range of potential means of control, including chemical fungicides [31,32,33,34], alternative compounds and basic substances (e.g., tannins, chitosan) [35,36,37,38,39], and the application of beneficial microorganisms such as *Bacillus* spp. [40,41,42]. However, the translation of these findings remains limited, as very few studies have evaluated their efficacy in the field [39].

The field of genomics research has so far yielded limited insights into *C. granati,* although a study was performed on its host during infection at the transcriptomic level [25]. There are genomic and transcriptomic resources available for some closely related *Coniella* spp., including two grape-infecting species (*C. vitis* [43] and *C. diplodiella* [44]) and the saprotrophic *C. lustricola* [45]. The prior genome-based studies that directly focused on *C. granati* were confined to ITS-based phylogenetic analyses, placing it within the order Diaporthales (family: Schizoparamaceae) [1]. Beyond the *Coniella* genus, other species of the order Diaporthales have been the subject of comprehensive genome sequencing projects, including *Botrytis* spp. (grey mould) with genome sizes of 43–55 Mb and ~12 K protein-coding genes [46]; *Colletotrichum gloeosporioides* (anthracnose) with a 62.8 Mb genome and 15,845 genes [47]; *Diaporthe* (syn. *Phomopsis*) *longicolla* (stem canker/dieback) with a 62 Mb genome and 16,597 genes [48]; and *Sclerotinia sclerotiorum* with a 38.8 Mb genome and 11,860 genes [49]. These genome projects provided valuable insights into the biology of these important fungal pathogens. Such findings have also been used to identify new targets for antifungal drugs and to develop new strategies for disease control.

Here, we present the first comprehensive genome analysis of *C. granati*, which is responsible for postharvest fruit rot and other diseases of pomegranate and other hosts. This foundational research paves the way for future investigations in the field of molecular plant pathology, ultimately enabling the development of effective strategies to control this destructive pathogen and safeguard pomegranate production.

## 2. Results

### 2.1. Genome Assembly

A total of 118,502 raw long reads were obtained for *C. granati* Ph1, comprising 12.47 Gb in total with an average read length of 105.2 Kb and maximum length of 242.7 Kb (Table S1). These were partitioned into 3,297,588 sub-reads comprising 12.23 Gb in total with an average length of 3708 bp and maximum of 187.8 Kbp (Table S1). Canu [50] generated 1,340,521 (5.3 Gb) corrected reads and assembled 1301 contigs totalling 46.8 Mb with 50% of this total length contained in 399 sequences (Table 1). The genome assembly was used as input to CATAStrophy—a bioinformatic method derived from the predicted CAZyme content of a genome [51]—to predict the infection mode of *C. granati* (Table 1). CATAStrophy predicted a combination of saprotroph (1), monomertroph (1), and extracellular mesotroph (0.94), which corresponded to saprotroph, biotroph, and hemibiotroph, respectively [51]. This is consistent with traditional hemibiotrophic classifications.

### 2.2. Analysis of Repetitive DNA

**Figure 1:**
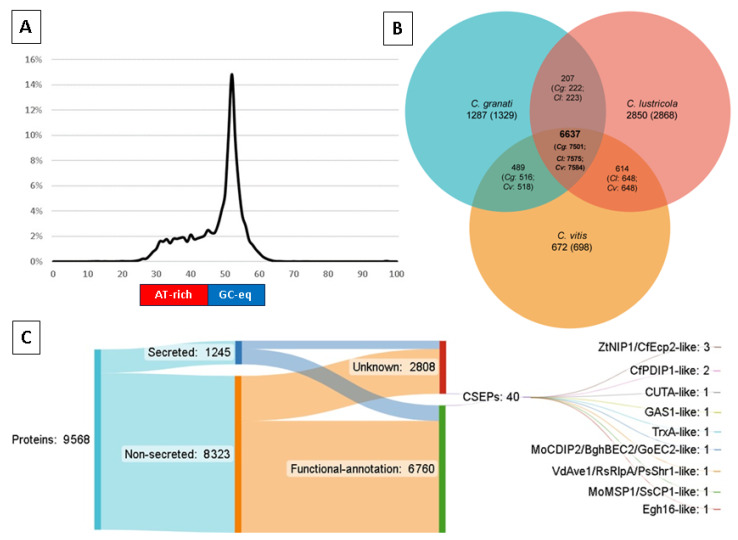
Summary of the genome features of *Coniella granati* Ph1. (**A**) The proportion of assembly length (y-axis) with consistent levels of G:C content indicated a high proportion of AT-rich, repetitive sequences relative to GC-equilibrated (GC-eq) regions. (**B**) Prediction of orthologous gene content comparing *C. granati* with sister species *C. lustricola* (saprotroph) and *C. vitis* (grape pathogen) indicated core and lineage-specific ortholog groups (gene numbers in parentheses). (**C**) Protein-coding gene prediction, functional annotation, and effector prediction in *Coniella granati* Ph1 revealed a minimal and functionally well-defined proteome with a relatively limited set of candidate secreted effector-like proteins (CSEPs).

The de novo prediction of repetitive DNA regions via TEtools (using repeatmodeller and repeatmasker) indicated that 24.5% of the genome assembly was repetitive, and the most numerous (15%) repeats were retrotransposons (Table 1, Data S1). The prediction of AT-rich regions of the genome assembly with OcculterCut indicated that almost one third (26.9%) of its genome was contained within gene-sparse and AT-rich compartments, which had G:C content ranging from ~30 to 45% (Figure 1A). The AT-rich regions defined by OcculterCut contained 660 loci at a density of 6.97 genes/Mbp compared to 277 genes/Mbp in G:C-equilibrated regions.

### 2.3. Annotation of Protein-Coding Genes and Their Putative Functions

Transcriptome sequencing was used to generate supporting evidence for exon features prior to performing the automated prediction of gene annotations. Of a total of 68,934,330 RNA-seq paired reads, 95.78% aligned to the genome assembly with HiSat2, with 11,536,471 pairs (33.47%) aligning concordantly exactly once, and 40,765,368 pairs (59.14%) aligning concordantly multiple times. A streamlined set of 9568 protein-coding gene annotations was predicted (Data S2, Data S3), the majority of which (9086 or 95%) were able to be assigned functional-annotation (Table 1, Figure 1C, Data S4). The prediction of extracellular secretion resulted in 1245 (13%) secreted proteins versus 8323 (87%) non-secreted proteins. Among conserved Pfam annotations, 71.3% (5933/8323) of non-secreted proteins matched one or more Pfam domains versus 66% (827/1245) of secreted proteins.

### 2.4. Coniella spp. Comparative Genomics

Due to the absence of an available proteome dataset for *C. vitis* QNYT13637, a predicted proteome was generated for this study (Data S5), which resulted in 9448 annotations within its 41.54 Mb assembly [43]. This was higher than its previously reported 7985 genes [43]; however, a similar number of 9403 genes had been reported for *C. diplodiella* within its 40.93 Mb assembly [44]. *C. diplodiella* comparisons were limited to its published genome metrics, as only unassembled reads were available at the time of writing [NCBI BioProject: PRJNA649095] [44] (Table 2). The *C. granati* assembly was of comparable total size (46.8 Mb versus 36.5–41.5 Mb), had poorer contiguity, had poorer estimated gene-content completeness, and had a far higher repeat content of 26.8% versus 5.8–12.7% for other *Coniella* spp. (Table 2).

Nucleotide-level whole-genome alignments of the *C. granati*, *C. lustricola*, and *C. vitis* assemblies indicated that *C. granati* had a higher proportion of its assembly conserved with that of *C. vitis* (Table 3A,B). Approximately one third of the *C. granati* assembly matched to the *C. vitis* assembly as opposed to 12% matching to the *C. lustricola* assembly. SNP variations detected between matching regions across the three species were observed to more frequently involve C↔T and A↔G transitions, which are typical of active repeat-induced point mutations (RIP) commonly observed in fungal lineages of the Pezizomycotina [52], totalling approximately 60% of all SNPs (Table 3C). All species appeared to exhibit similar levels of RIP-like variation relative to each other across conserved regions.

Gene-level comparisons of predicted orthology between *C. granati*, *C. lustricola*, and *C. vitis* indicated a core set of 6637 ortholog groups (that included 7501 *C. granati* genes) (Figure 1B). There were 1287 ortholog groups specific to *C. granati*, which was less than *C. lustricola* (2850) and more than *C. vitis* (672). *C. granati* shared 207 ortholog groups with *C. lustricola* and 489 groups with *C. vitis*, which was comparable to the 614 groups shared between the two sister species but not observed in *C. granati*.

### 2.5. Prediction of Pathogenicity Genes

**Figure 2:**
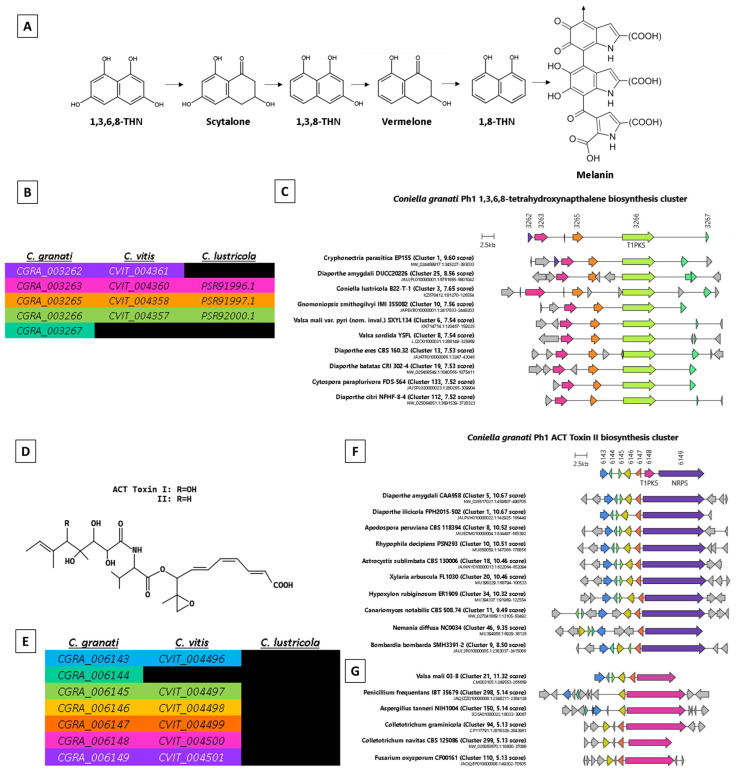
Summary of two highly conserved secondary metabolite biosynthesis gene clusters of the *Coniella granati* Ph1 genome assembly. (**A**) Chemical structure of 1,3,6,8 tetrahydroxynapthalene (1,3,6,8-THN), which is a precursor of melanin. (**B**) Contiguous clusters of orthologs matching the 1,3,6,8-THN cluster of *C. granati* Ph1 versus other *Coniella* spp. (**C**) The top 10 cluster predictions matching the predicted 1,3,6,8-THN synthesis cluster predicted in the *C. granati* Ph1 assembly, excluding duplicate species and genus level taxa. (**D**) Chemical structure of ACT Toxin II. (**E**) Contiguous clusters of orthologs matching the ACT-Toxin II cluster of *C. granati* Ph1 versus other *Coniella* spp. (**F**) The top 10 cluster predictions matching the predicted ACT-Toxin II synthesis cluster predicted in the *C. granati* Ph1 assembly, excluding duplicate species and genus level taxa, with one or more matches to the putative non-ribosomal peptide synthase (NRPS) locus (**F**) or the type 1 polyketide synthase (T1PKS) locus (**G**).

The prediction of Candidate Secreted Effector-like Proteins (CSEPs), which required predicted secretion and a Predector score ≥ 1.5, resulted in a relatively small set of 40 CSEPs, all but four of which were cysteine-rich (2–12 residues) (Table 4, Data S6). These CSEPs were located within 23.9 Kb (6.3 Kb on average) of an AT-rich region or contig end (which are presumed to be repeat-rich, Table 2, Figure S1, Data S7). The prediction of secondary metabolite synthesis clusters (SMCs) with AntiSMASH indicated 36 clusters (Table S2). Two SMC clusters contained genes with identical matches to loci involved in the production of the secondary metabolites (SMs) 1,3,6,8-tetrahydroxynapthalene (1,3,6,8-THN) (Figure 2A) and ACT-Toxin II (Figure 2D). Other SMs potentially produced by *C. granati* may also include burnettramic acid A, depudecin, ascochlorin, squalestatin S1, and chaetoglobosin; however, these matches were less reliable and ranged from 33 to 44% (Table S2).

Further analysis of the two highly conserved gene clusters potentially involved in the synthesis of 1,3,6,8-THN and ACT-Toxin II was performed with CAGECAT (Data S8). The 1,3,6,8-THN cluster (Figure 2A–C) contained *C. granati* loci *CGRA_003262-003267* with *CGRA_003266* predicted to encode a type 1 polyketide synthase (T1PKS). Orthology comparisons to *Coniella* spp. corresponded to *C. vitis CVIT_004361-004357* (missing an ortholog for *CGRA_003267*) and to *C. lustricola PSR91996.1 -92000* (missing orthologs to *CGRA_003262* and *CGRA_003267*) (Figure 2B). CAGECAT also predicted a *C. lutsricola* cluster in sequence KZ678412.1 [81270-126554] but was not able to search against *C. vitis* due to its protein annotations being unavailable to the NCBI-protein nr database at the time of writing. Highest cluster conservation was observed with the Chestnut Blight pathogen *Cryphonectria parasitica* EP155, but clusters within the genomes of many other plant- and/or fruit-pathogen species were also indicated (Figure 2). Within the ACT-Toxin II biosynthesis cluster (Figure 2D–G), *CGRA_006143* was predicted to encode an NRPS protein, and *CGRA_006144* was predicted to encode a T1PKS (possibly truncated relative to homologs, Figure 2G). This cluster was notably absent from the non-pathogenic sister species *C. lustricola*, but it was predicted to be conserved in the grape pathogen *C. vitis* (matching *CVIT_004496-4501* but missing an ortholog of the T1PKS *CGRA_006144*). The cluster was also highly conserved across several fungal species, many of which were plant- and/or fruit-pathogen species (Figure 2F–G), including *Diaporthe amygdali* (soybean, almond, grapevine, and blueberry) and *Diaporthe illicola* (holly). Predominantly, these cluster matches corresponded to the NRPS locus and lacked the T1PKS (Figure 2F); however, variants were also detected in some species containing the T1PKS and lacking the NRPS locus (Figure 2G).

## 3. Discussion

Pomegranates have been cultivated since ancient times, and the crop is currently expanding quickly [21,53]. Because of their high polyphenol content, pomegranates are considered as functional foods, which has increased global demand for fresh fruit in recent years [53]. However, following harvest, fruit quality drops due to fungal infections, which could endanger pomegranate marketability [54]. The pathogenic fungus *C. granati* has been identified as one of the main causal agents of postharvest decay in pomegranates, which minimises the fruit market value [55]. Specifically, this pathogen results in significant postharvest losses that may reach 30% and causes symptoms in plants and fruits such as collar rot, leaf spot, and fruit decay [21]. Nevertheless, there is little information available regarding this host–pathogen interaction and pathogen epidemiology [21] despite its significance.

A high proportion of the *C. granati* Ph1 genome assembly comprised AT-rich and repetitive regions spanning over a quarter of the total assembly length. Repeats were widely interspersed throughout the genome at high frequency and posed a significant barrier to effective chromosome-level assembly even using long-read sequencing approaches. Indeed, of all the *Coniella* species subjected to whole-genome analysis so far, *C. granati* assembly is the poorest in terms of contiguity, but correspondingly, it has the highest repetitive DNA content. Repeat-rich regions in *C. granati* were gene-sparse and AT-rich, non-homologous to other *Coniella* spp.; they were duplicated in high frequency throughout the genome and tended to occur either throughout the entire contig or at termini. Despite technical obstacles for genome assembly, analysis of the protein-coding gene content of *C. granati* Ph1 was comparatively simple with the genome assembly found to encode an extremely minimalistic proteome relative to most Ascomycetes [56] (but comparable to other *Coniella* spp.), and a surprisingly small set of 40 candidate secreted effector-like proteins (CSEPs).

The 40 CSEPs were all located near AT-rich and repetitive regions that would be targeted by RIP. This would likely place the CSEPs (and many other genes) within the range for the leakage of repeat-targeted RIP mutations into neighbouring non-repetitive sequences, which has been previously established as important for the rapid adaptation of avirulence in other hemibiotrophs [57]. ‘RIP-leakage’ is a genome mutagenesis process involving the pseudogenisation of non-repetitive genes located near repeat regions by RIP. This can introduce nonsense mutations that lead to early stop codons, generating avirulent loss-of-function mutants that may avoid PAMP-triggered immunity in the host [57].

In addition to the small size of the *C. granati* CSEP set, many CSEPs were either functionally annotated or were homologous to well-characterised effectors from other hemibiotrophs (Table 2, Data S4). Three CSEPs were homologs of the necrosis-inducing ZtNIP1/CfEcp2 effector [58,59], and one of these was also predicted to localise to the chloroplast. Furthermore, there were two homologs of CfPDIP1 [60], which may trigger the hypersensitive response (HR). Other effector homologs included MoCDIP2, which is localised to the mycelia and appressoria and induces cell-death [61], the virulence-associated cutinase CUTA [62], the antimicrobial virulence factor VdAve1/RsRlpA/PsShr1 [63], the cerato-platanin MoMSP1/SsCP1 [64,65], and thioredoxin TrxA [66]. Of the 40 CSEPs, 28 were also predicted to have orthologs in sister species *C. lustricola* and *C. vitis*, potentially indicating core conservation across the *Coniella* genus. The majority of CSEPs with homology to known effectors or with other functional annotations belonged to this type. The remainder comprised four orthologs shared with *C. vitis* and missing from *C. lustricola,* three orthologs shared with *C. lustricola* and missing from *C. vitis*, and five orthologs missing from both sister species. The five *C. granati*-specific CSEPs included two with unknown function with the others each matching to thioredoxin, carboxylesterase, and MutS DNA mismatch repair domains (Table 4).

Overall, the relatively small set of CSEPs with credible and well-studied homologs in other hemibiotroph species presented an opportunity to infer the potential mechanisms of *C. granati* host–pathogen interactions. Additionally, the prediction of highly conserved SMCs potentially involved in the production of 1,3,6,8-THN and ACT-Toxin II also provided further clues toward understanding the virulence mechanisms of *C. granati*. 1,3,6,8-THN is a melanin precursor [67] (Figure 2A) and may have a pathogenicity-related role in fungal cell-wall strengthening [68]; however, similar clusters were observed across a range of species including the non-pathogenic *C. lustricola*. The conserved SMC putatively involved in ACT-Toxin II synthesis is more compelling due to its absence in *C. lustricola*. ACT-Toxin II was originally described for the tangerine pathotype of *Alternaria alternata* (ACT = *Alternaria citri* Tangerine) [69], where it is required for tangerine infection and has since been reported to play an important role in the virulence of other pomegranate-infecting species including *Talaromyces albobiverticillius* (pomegranate pulp rot) [70]. Its high level of conservation across a broad range of plant–pathogen species (Figure 2F) supports a common role in the infection of a variety of plant fruiting structures.

## 4. Materials and Methods

### 4.1. Sample Collection and Culture

The strain of *C. granati* sequenced in the present study (Ph1) was isolated from pomegranate fruit of cv. Wonderful collected in a packing house in Apulia (southern Italy). Fruit showed circular brownish–yellow lesions, beginning in the crown area, quickly expanding to entire fruit, with softening of the tissues including arils. This isolate was identified according to morphological microscopic features of hyphae and conidia as well as the sequence of the ITS1-5.8S-ITS2 region of the rDNA, which was identical to reference sequences [9]. Fungal DNA was extracted using the Quick-DNA™ Fungal/Bacterial Miniprep Kit (Zymo Research, Irvine, CA, USA) from 100 mg of 10-day-old mycelium of Ph1 strain growing on potato dextrose agar (PDA) Petri dishes.

### 4.2. Genome Sequencing and Assembly

Fungal genomic DNA was sequenced by 150 bp paired-end (PE) reads through the Illumina Novaseq 6000 platform (Illumina, San Diego, CA, USA) using the Novogene NGS DNA Library Prep Set (Cat No.PT004) (Novogene, Beijing, China) for library construction as well by long reads through the PacBio SMRTbell sequel II platform (CLR mode) (Pacific Biosciences of California Inc., Menlo Park, CA, USA). Read correction and de novo assembly was performed with Canu v2.2 (genomeSize = 40 m; minOverlapLength = 300; corMaxEvidencErate = 0.15) [50].

### 4.3. Transcriptome Sequencing for Gene Annotation Supporting Evidence

Transcriptomic mRNA was extracted in triplicate from the mycelial phase grown on PDA medium from a fresh 10-day-old culture using the Quick-RNA™ Fungal/Bacterial Miniprep kit (Zymo Research, Irvine, CA, USA). RNA-seq reads were generated via Illumina (PE150 Novaseq 6000) using the Novogene NGS RNA Library Prep Set (PT042) (Novogene, Beijing, China). Transcriptomics PE reads were aligned to the genome assembly with Hisat2 v2.2.1 (max-intronlen 5000; dta) [71] to assist in the annotation of protein-coding gene regions. Transcriptome-aligned genome regions were determined with StringTie v2.2.1 [72] and used as input to FunAnnotate v1.8.15 (predict; max_intronlen 1000) [73] to support automated gene annotation.

### 4.4. Prediction of DNA Repeats, Protein-Coding Genes and Gene Functions

Repetitive sequences were predicted using Dfam TE Tools 1.88 [38]. Genome-wide G:C compartmentalisation and AT-rich regions were predicted with OcculterCut v1.1 [52] using the Canu assembly and Funannotate GFF3 annotation as inputs. Functional annotations were predicted via Interproscan v5.63-95.0 [74] and effector-like properties—including predicted secretion—were predicted via Predector v1.2.7 [75]. Infection mode (trophic type) was predicted by CATAStrophy v0.1.0 (HMMER 3.3 vs dbCAN v10) [51]. Secondary metabolite clusters (SMCs) were predicted with antiSMASH v6.1.1 [76], and highly conserved SMCs were further analysed for conservation in other fungal species via CAGECAT (release 1.0, “Fungi[ORGN]”) [77,78,79].

### 4.5. Comparative Genomics between Coniella spp.

Genome assembly sequences of alternate *Coniella* spp. were obtained from the NCBI Genome database for *C. vitis* isolate QNYT13637 [NCBI Genome: GCA_011317545.1] [43] and *C. lustricola* B22-T-1 [NCBI Genomes: GCA_003019895.1] [45]. The *C. vitis* and *C. lustricola* assemblies were aligned to the *C. granati* Ph1 assembly with MUMMER v3.23 (nucmer-mum) [80]. Protein-coding gene annotations were obtained for *C. lustricola* from the same source as above; however. as *C. vitis* gene annotations were not available, a new dataset was generated for this study (Data S5). Comparisons to *C. diplodiella* in this study were limited published genome metrics, as only unassembled reads were available at the time of writing [BioProject: PRJNA649095] [44]. MUMmer v3.23 was used to align genome assemblies (nucmer–maxmatch) and summarise whole-genome alignment metrics (dnadiff) [81]. Funannotate 1.8.15 [73] was used to predict missing gene annotations for *C. vitis* with translated annotations of *C. graniti* and *C. lustricola* provided as supporting data. Predicted proteomes of *C. granati*, *C. lustricola*, and *C. vitis* were clustered into ortholog groups (including paralogs and singleton groups) with ProteinOrtho6 (--selfblast, --singletons) [82]. Exome completeness metrics were sourced from prior studies or estimated for *C. granati* and *C. lustricola* using BUSCO (v5.5.0 genome, auto-lineage, metaeuk) [83].

## 5. Conclusions

These genomic resources and CSEP predictions presented in this study are important foundational data for subsequent genomic and molecular plant pathology studies for the pomegranate pathogen *C. granati*. Its minimally-encoding and highly repeat-dispersed genome represents an interesting ‘edge case’ among most fungal plant-pathogens and could provide future insights in comparative genomics studies versus other species with similar genomic features and long latent phases [51,84]. Despite a minimalistic proteome and relatively smaller CSEP set than is typically reported among plant pathogenic fungi, *C. granati* is an effective pathogen of pomegranate. Future improvement of this initial genome resource and additional pan-genome sequencing may reveal the nature and extent of genomic variation between isolates of *C. granati* and how this pathogen may adapt to changing host- and control-based selection pressures. As biotroph and hemibiotroph populations may only require a small number of avirulence effectors capable of periodic RIP-mediated pseudogenisation in response to host R-gene recognition [57], we speculate that this may have influenced the ‘streamlining’ of the protein-coding gene and CSEP contents of the *C. granati* genome over time.

## Figures and Tables

**Table 1:** Genome assembly (A), repetitive DNA (B), and protein-coding annotation (C) metrics for the genome of *Coniella granati*.

(A) Genome assembly	
Total	46.8 Mb/46,832,344 bp
Sequence number	2009
N50 length	23,311 bp
N50 number	399
Max length	220,902 bp
Mean length	35,815 bp
(B) Repetitive DNA	
Proportion of genome in AT-rich regions	26.9%
Repetitive DNA	11.69 Mb (24.95%)
Retroelements	15%
LINEs	0.58%
LTR elements	14.42%
Copia-like	3.34%
Gypsy-like	11.08%
DNA transposons	1.07%
Unclassified	6.94%
Low complexity/small RNA/Simple repeats	1.93%
(C) Predicted Pathogenicity Features	
CATAStrophy-predicted infection type	Hemibiotrophic: extracellular mesotroph (0.94); monomertroph (1); saprotroph (1);
Protein-coding genes	9568
Functionally-annotated proteins	9086 (95%)
Secreted proteins	1245 (13%)
Candidate pathogenicity effectors (Predector ≥ 1.5)	40

**Table 2:** Comparison of the genome assembly metrics of *C. granati* versus reported metrics from published genome studies of sister species *C. lustricola*, *C. vitis*, and *C. diplodiella*.

Feature	*C. granati*	*C. lustricola*	*C. vitis*	C. diplodiella *
Host range	Pomegranate	Saprophyte	Grapevine	Grapevine
Isolate	Ph1	B22-T-1	QNYT13637	WR01
Assembly size (Mb)	46.83	36.56	41.54	40.93 *
Sequence number	2009	634	22	13 *
N50 number	399	76	5	*
N50 length (Mb)	0.02	0.14	3.20	3.99
Repetitive (%)	26.9	*	5.76	12.74
Gene annotations	9568	11,317	7985 (published) * 9448 (this study)	9403 *
BUSCO % completeness	84.8%	96.0%	99.3%	97.6%

* data not available.

**Table 3:** Comparisons of predicted genome assembly features between *Coniella* spp. (*C. granati* (Cg)*, C. lustricola* (Cl) and *C. vitis* (Cv)) derived from MUMmer (nucmer) alignments and summarised by dnadiff: (A) the percentage of total sequences conserved between species; (B) the percentage of total bases aligned between species; and (C) the number and percentage of SNP mutations in alignments between species.

(A) % sequences aligned (x vs. y)	*C. granati*	*C. lustricola*	*C. vitis*
*C. granati*	-	64.3%	78.3%
*C. lustricola*	84.54	-	85.8%
*C. vitis*	100%	95.45%	-
(B) % bases aligned (x vs. y)	*C. granati*	*C. lustricola*	*C. vitis*
*C. granati*	-	12%	33.48%
*C. lustricola*	15.4%	-	15.4%
*C. vitis*	40%	14.5%	-
(C) SNPs (number and %total)	Cg-vs.-Cl	Cg-vs.-Cv	Cl-vs.-Cv
A-C	35,949 (4.9%)	81,114 (4.9%)	33,220 (4.5%)
A-G *	122,165 (16.5%)	279,243 (16.8%)	111,223 (15%)
A-T	23,446 (3.2%)	51,250 (3.1%)	23,029 (3.11%)
C-A *	32,134 (4.3%)	72,791 (4.4%)	34,545 (4.7%)
C-G	47,737 (6.4%)	92,555 (5.6%)	50,542 (6.8%)
C-T *	109,902 (14.9%)	254,516 (15.3%)	117,764 (15.9%)
G-A *	109,974 (14.9%)	254,462 (15.3%)	115,929 (15.7%)
G-C	46,769 (6.3%)	91,853 (5.5%)	50,407 (6.8%)
G-T	31,558 (4.3%)	73,048 (4.4%)	34,621 (4.7%)
T-A	23,339 (3.1%)	51,022 (3.1%)	22,848 (3.1%)
T-C *	121,043 (16.4%)	277,912 (16.7%)	111,806 (15.1%)
T-G	35,746 (4.8%)	80,624 (4.9%)	33,452 (4.5%)

* Repeat-induced point mutation (RIP)-like SNPs.

**Table 4:** Supporting evidence for Candidate Secreted Effector-like Proteins (CSEPs) of *Coniella granati* predicted by Predector (secreted and score ≥ 1.5). The length of encoded proteins (aa), number of cysteine residues (#Cys), and the distance of each locus to the contig or scaffold end as well as whether the locus resided in an AT-rich (AT) or <25 Kb of a sequence end (End) region is also indicated.

Locus	PAV *	Score	Effector Homology and Functional Annotations	#Cys	Len (aa)	Distance (bp)	Region Type
PGRA_006204	Core	3.086	Homology:CfEcp2,ZtNIP1; Pfam:PF14856(Hce2);	4	227	9970	End
PGRA_007290	Cg-Cl	2.646	[No match]	7	141	3056	AT
PGRA_008218	Core	2.505	Homology: ZtNIP1,CfEcp2; Pfam:PF14856(Hce2); Localiser:chloroplast;	5	197	782	End
PGRA_002694	Cg-Cv	2.474	[No match]	0	228	23,880	End
PGRA_001911	Core	2.443	[No match]	4	170	18,286	End
PGRA_008765	Core	2.427	[No match]	12	137	3446	End
PGRA_009449	Core	2.398	PHIbase:CUTA(KO-unaffected pathogenicity); Pfam:PF01083(Cutinase)	6	195	22	End
PGRA_006664	Core	2.339	PHIbase: GAS1(KO-reduced virulence); Pfam:PF11327(Egh16-like); Localiser:nucleus;	4	257	1446	AT
PGRA_002414	Core	2.299	[No match]	4	224	13,631	AT
PGRA_004539	Cg	2.162	Pfam:PF00488(MutS_V);	2	249	2890	AT
PGRA_008027	Cg-Cv	2.138	[No match]	12	184	26	AT
PGRA_003492	Cg-Cl	2.134	Localiser:nucleus;	8	181	5414	End
PGRA_008885	Core	2.095	Pfam:PF14273(DUF4360);	4	217	4169	End
PGRA_008860	Cg-Cv	2.092	[No match]	6	120	2506	AT
PGRA_009224	Core	2.084	Homology: CfPDIP1;	6	113	2644	End
PGRA_007015	Core	2.068	Homology: CfPDIP1;	8	130	7150	End
PGRA_008976	Cg	2.045	PHIbase:TrxA,Thioredoxin_1(KO-reduced virulence); Pfam:PF00085(Thioredoxin);	5	137	74	End
PGRA_001981	Core	2.044	Pfam:PF11327(Egh16-like);	4	239	7609	End
PGRA_004164	Cg-Cl	2.016	Homology: GoEC2,MoCDIP2,BghBEC2; Pfam:PF05730(CFEM); Localiser:nucleus;	7	154	255	End
PGRA_005485	Core	1.994	Homology:ZtNIP1,CfEcp2; Pfam:PF14856(Hce2);	4	167	95	End
PGRA_001358	Core	1.982	Pfam:PF06766(Hydrophobin_2);	8	97	23,442	AT
PGRA_006064	Core	1.953	Localiser:nucleus;	8	143	6677	End
PGRA_007124	Cg-Cv	1.936	Pfam:PF01822(WSC);	7	125	7176	AT
PGRA_009571	Cg	1.911	[No match]	6	75	35	AT
PGRA_005443	Core	1.908	[No match]	9	194	5754	End
PGRA_001444	Core	1.898	[No match]	8	127	17,504	End
PGRA_005889	Core	1.892	[No match]	8	196	14,164	End
PGRA_008276	Core	1.869	[No match]	0	101	708	AT
PGRA_009699	Cg	1.864	Pfam:PF00135(COesterase);	2	171	245	End
PGRA_002373	Core	1.79	Pfam:PF00085(Thioredoxin);	2	175	17,357	End
PGRA_001309	Core	1.762	Pfam:PF01161(PBP);	10	156	22,013	AT
PGRA_008695	Cg	1.695	[No match]	1	204	2994	End
PGRA_008501	Core	1.647	[No match]	9	212	2852	End
PGRA_000088	Core	1.644	Pfam:PF01083(Cutinase);	5	253	6346	AT
PGRA_000913	Core	1.628	Pfam:PF01105(EMP24_GP25L);	3	222	0	AT
PGRA_008930	Core	1.623	Pfam:PF10270(MMgT);	1	139	1778	End
PGRA_002139	Core	1.59	[No match]	7	207	440	AT
PGRA_004189	Core	1.577	Pfam:PF11327(Egh16-like);	4	244	14,924	End
PGRA_009219	Core	1.534	Homology: RsRlpA,VdAve1,PsShr1;	5	132	68	AT
PGRA_006644	Core	1.521	Homology: SsCP1,MoMSP1	10	221	189	AT

* Predicted conservation across *Coniella* spp., based on presence–absence variation (PAV) in orthology comparison between *C. granati* (Cg), *C. lustricola* (Cl), and *C. vitis* (Cv); #Cys = cysteine residues.

## Data Availability

The genome and transcriptome sequencing datasets generated for this study can be found under NCBI BioProject record PRJNA1130623.

## Supplementary Materials

The following supporting information can be downloaded at https://www.mdpi.com/article/10.3390/ijms25189997/s1.
