# Supplementary material for: Highly Repetitive Genome of Coniella granati (syn. Pilidiella granati), the Causal Agent of Pomegranate Fruit Rot, Encodes a Minimalistic Proteome with a Streamlined Arsenal of Effector Proteins
Source: Int J Mol Sci. 2024 Sep 17;25(18):9997. doi: 10.3390/ijms25189997 (PMC11432717; doi:10.3390/ijms25189997)
Supplement: Supplementary file 1 [file ijms-25-09997-s001.zip › supplementary_figures_tables_combined.pdf]

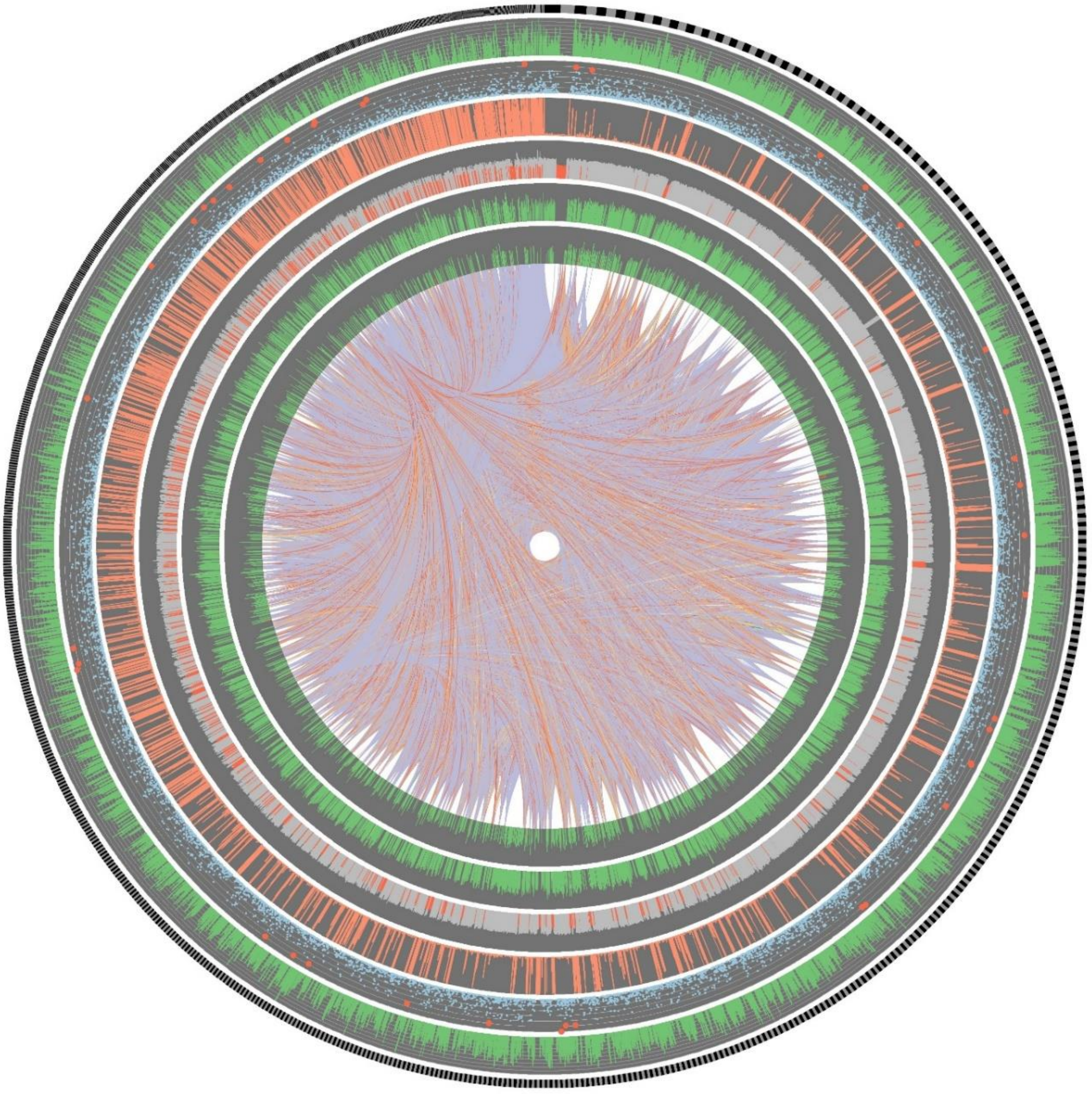

**Figure S1.** Circular overview of the highly-repetitive *Coniella granati* Ph1 genome assembly, with contigs indicated in black and grey on the outer track, and showing various gene and repeat-based features within inner tracks: A) percent density of genes within windows of up to 50 kb (green); B) Predector scores from -3 to 3 indicating likelihood of genes encoding pathogenicity effector proteins (light blue, with scores >1.5 in red); C) percent density of repeats within windows of up to 50 kb (red); D) G:C percentage within 10 kb windows (green, with AT-rich regions i.e. <40% G:C in red); E) homology to alternate *Coniella* spp. (green, from 75-100%, with <80% highlighted red) for *C. vitis* (outer) and *C. lustricola* (inner), and; F) interchromosomal homologous sequences of > 2kb (color scale indicates shorter=blue or longer=red matches);

Table S1: DNA sequence read and genome assembly metrics

| Type | Raw | sub-reads | canu-corrected | canu-assembly |
|---|---|---|---|---|
| **Number** | 118,502 | 3,297,588 | 1,340,521 | 1,301 |
| **Total** | 12,466,672,316 | 12,229,538,286 | 5,305,472,563 | 46,832,344 |
| **Mean** | 105,202 | 3,708 | 3,957 | 35,815 |
| **Max** | 242,748 | 187,882 | 187,882 | 220,902 |
| **N50** | 161,966 | 4,066 | 4,272 | 23,311 |

Table S2: Summary of secondary metabolite synthesis clusters (SMC) predicted by AntiSMASH

| Sequence | SMC type | Start | End | Predicted products | Predicted Role | Similarity |
|---|---|---|---|---|---|---|
| tig0022 | T1PKS,NRPS | 61,336 | 116,837 | burnettramic acid A | Alkaloid + Polyketide:Iterative type I | 44% |
| tig0032 | T1PKS | 1,539 | 51,861 | | | |
| tig0098 | NRPS-like | 1 | 29,705 | | | |
| tig0100 | NRPS-like | 1 | 10,029 | | | |
| tig0106 | T1PKS | 15,439 | 63,062 | | | |
| tig0143 | T1PKS | 1 | 38,815 | depudecin | Polyketide:Iterative type I | 33% |
| tig0144 | T3PKS | 5,564 | 47,137 | | | |
| tig0158 | NRPS-like | 2,525 | 45,740 | | | |
| tig0161 | terpene | 11,225 | 33,424 | | | |
| tig0201 | T1PKS | 23,164 | 63,743 | 1,3,6,8-tetrahydroxynaphthalene | Polyketide | 100% |
| tig0223 | T1PKS | 18,005 | 46,024 | | | |
| tig0227 | Other | 1 | 28,830 | | | |
| tig0248 | NRPS,NRPS-like | 1 | 46,980 | | | |
| tig0321 | NRPS | 1 | 44,401 | | | |
| tig0342 | NRPS-like | 1 | 28,517 | | | |
| tig0357 | NRPS-like | 1 | 35,170 | | | |
| tig0449 | NRPS | 1 | 29,717 | | | |
| tig0547 | T1PKS,NRPS | 1 | 28,761 | ACT-Toxin II | Polyketide | 100% |
| tig0550 | T1PKS | 1 | 23,395 | | | |
| tig0658 | NRPS | 1 | 29,707 | | | |
| tig0712 | NRPS,T1PKS | 1 | 29,757 | | | |
| tig0740 | NRPS | 1 | 15,679 | | | |
| tig0764 | NRPS-like | 1,489 | 26,247 | | | |
| tig0800 | NRPS-like | 1 | 28,540 | | | |
| tig0872 | T1PKS | 1 | 19,526 | ascochlorin | Terpene + Polyketide | 37% |
| tig0915 | T1PKS | 1 | 15,375 | | | |
| tig0993 | T1PKS | 1 | 15,974 | | | |
| tig1066 | NRPS | 1 | 18,910 | | | |
| tig1119 | T1PKS,NRPS | 1 | 16,423 | | | |
| tig1247 | terpene | 1 | 13,889 | squalestatin S1 | Terpene | 40% |
| tig1278 | NRPS-like | 1 | 10,026 | | | |
| tig1448 | NRPS-like | 1 | 6,122 | | | |
| tig1965 | NRPS,T1PKS | 1 | 51,270 | chaetoglobosins | NRP + Polyketide:Iterative type I | 42% |
| tig1966 | T1PKS | 1 | 35,834 | | | |
| tig2007 | T1PKS | 1 | 25,669 | | | |
| tig2042 | T1PKS | 1 | 24,170 | | | |
