# Supplementary material for: Highly Repetitive Genome of Coniella granati (syn. Pilidiella granati), the Causal Agent of Pomegranate Fruit Rot, Encodes a Minimalistic Proteome with a Streamlined Arsenal of Effector Proteins
Source: Int J Mol Sci. 2024 Sep 17;25(18):9997. doi: 10.3390/ijms25189997 (PMC11432717; doi:10.3390/ijms25189997)
Supplement: Supplementary file 1 [file ijms-25-09997-s001.zip › DataS8_CAGECAT.zip!/1368_THN.zip!/results/V161L934Z753T99_plot.html]

cblaster


cblaster

---

If you found cblaster useful, please cite:

```
						Gilchrist, C. L., Booth, T. J., van Wersch, B., van Grieken, L., Medema, M. H., & Chooi, Y. H. (2021).
						cblaster: a remote search tool for rapid identification and visualization of homologous gene clusters.
						Bioinformatics Advances, 1(1), vbab016.
					
```

---

Click clusters to hide rows, or queries to hide columns.
The original view can be restored using the reset filters button.

Save SVG
Reset filters


---

Toggle visibility of plot elements:

Hit counts
Multi-hit cell borders
Genomic coordinates


---

Toggle positions of plot elements:

Multi-line cluster information
Query sequence names


---

Adjust size of plot elements:

Cell width:
px

Cell height:
px
